# Knowledge and attitudes towards influenza and influenza vaccination among pregnant women in Kenya

**DOI:** 10.1016/j.vaccine.2020.08.015

**Published:** 2020-10-07

**Authors:** Nancy A. Otieno, Bryan Nyawanda, Fredrick Otiato, Maxwel Adero, Winnie N. Wairimu, Raphael Atito, Andrew D. Wilson, Ines Gonzalez-Casanova, Fauzia A. Malik, Jennifer R. Verani, Marc-Alain Widdowson, Saad B. Omer, Sandra S. Chaves

**Affiliations:** a Kenya Medical Research Institute, Center for Global Health Research, Kisumu, Kenya; b Emory University Rollins School of Public Health, Hubert Department of Global Health, Atlanta, GA, USA; c Centers for Disease Control and Prevention, Division of Global Health Protection, Kenya; d Centers for Disease Control and Prevention, National Center for Immunization and Respiratory Diseases, Kenya

**Keywords:** Pregnant women, Influenza vaccine, Knowledge, Attitudes

## Abstract

**Background:**

Influenza vaccination during pregnancy benefits mothers and children. Kenya and other low- and middle-income countries have no official influenza vaccination policies to date but are moving towards issuing such policies. Understanding determinants of influenza vaccine uptake during pregnancy in these settings is important to inform policy decisions and vaccination rollout.

**Methods:**

We interviewed a convenience sample of women at antenatal care facilities in four counties (Nairobi, Mombasa, Marsabit, Siaya) in Kenya. We described knowledge and attitudes regarding influenza vaccination and assessed factors associated with willingness to receive influenza vaccine.

**Results:**

We enrolled 507 pregnant women, median age was 26 years (range 15–43). Almost half (n = 240) had primary or no education. Overall, 369 (72.8%) women had heard of influenza. Among those, 288 (78.1%) believed that a pregnant woman would be protected if vaccinated, 252 (68.3%) thought it was safe to receive a vaccine while pregnant, and 223 (60.4%) believed a baby would be protected if mother was vaccinated. If given opportunity, 309 (83.7%) pregnant women were willing to receive the vaccine. Factors associated with willingness to receive influenza vaccine were mothers’ belief in protective effect (OR 3.87; 95% CI 1.56, 9.59) and safety (OR 5.32; 95% CI 2.35, 12.01) of influenza vaccines during pregnancy.

**Conclusion:**

Approximately one third of pregnant women interviewed had never heard of influenza. Willingness to receive influenza vaccine was high among women who had heard about influenza. If the Kenyan government recommends influenza vaccine for pregnant women, mitigation of safety concerns and education on the benefits of vaccination could be the most effective strategies to improve vaccine acceptance.

## Highlights

• Willingness to be vaccinated against influenza while pregnant was high.
• Willingness to vaccinate was higher among pregnant women who knew about influenza.
• Government recommendation for the vaccine may lead to higher rates of acceptance.
• Improving disease knowledge and mitigating safety concerns may improve acceptance.
• Hesitant mothers should be educated on benefits of the vaccine during pregnancy.

## 1 Introduction

Influenza virus infection during pregnancy has been associated with increased influenza-related morbidity and mortality [1], [2]. In tropical settings influenza viruses circulate year-round [3], and several studies have reported influenza-associated disease burden that is similar or higher than estimates from temperate countries [4], [5], [6], [7], [8], [9]. In Kenya, influenza viruses circulate nearly in all months of the year with increased activity between July and November [4]. Published data on the burden of influenza in pregnant women in Africa remain scarce. However, data from South Africa demonstrated that pregnant women have increased risk of influenza-associated hospitalization [10]. Moreover, human immunodeficiency virus (HIV) and active tuberculosis infections are important risk factors for severe disease and death among influenza cases [11], [12]; thus pregnant women in sub-Saharan Africa could be at greater risk of severe influenza-associated disease outcomes.

The World Health Organization (WHO) Strategic Advisory Group of Experts (SAGE) on immunization recommended pregnant women as the highest priority group for influenza vaccination [13], based on compelling evidence on safety and effectiveness of the vaccine in protecting both pregnant women and young infants (through passive antibody transfer) against influenza disease and associated severe outcomes [14], [15], [16], [17], [18]. Rates of influenza-associated hospitalizations are highest during the first six months of life, age in which no influenza vaccines are currently licensed [19]. Despite the recommendation to prioritize pregnant women for influenza vaccination, many countries are yet to make official recommendations; and in those where influenza vaccination is recommended during pregnancy, uptake of the vaccine remains suboptimal [20], [21], [22]. Underestimation of the potential severity of influenza virus infection, misconceptions of vaccine safety by pregnant women, and failure of obstetric health care providers to recommend and provide the vaccine to pregnant women have been cited as some of the leading reasons for low uptake of influenza vaccines during pregnancy in most middle- and high-income countries [23].

Understanding determinants of influenza vaccine acceptance during pregnancy in low- and low to middle-income settings is important to inform rollout of maternal influenza vaccination programs and guide strategies to promote uptake of the vaccine. Tetanus toxoid is the only recommended maternal vaccine in Kenya. Although influenza vaccines are available in the private sector, the number of doses ordered annually is limited. We sought to understand knowledge and attitudes of Kenyan pregnant women on influenza vaccination and factors that would influence their willingness to receive influenza vaccines during pregnancy to inform decision making on future plans to expand the pregnancy vaccination platform in the country.

## 2 Methods

### 2.1 Study sites and study population

This analysis was part of a larger cross-sectional survey examining factors that shape acceptance of maternal vaccines in Kenya as a collaboration among the Kenyan Medical Research Institute (KEMRI), Emory University in Atlanta, Georgia, and the Centers for Disease control and Prevention (CDC)- Kenya. Study sites were Nairobi, Mombasa, Siaya and Marsabit counties; site choice was guided by geographic representation, cultural and religious diversity, aiming to achieve a mix of urban, *peri*-urban, and rural settlements in Kenya. Pregnant women were recruited from seven healthcare facilities, including the main public referral hospitals for Mombasa, Siaya and Marsabit counties, two additional public facilities in Mombasa and Nairobi counties, and two private non-profit facilities: Tabitha Clinic (located in Kibera, an informal urban settlement in Nairobi) and St. Elizabeth Lwak Mission Hospital (located in rural Siaya County). The latter two facilities are non-governmental organizations providing primary care to underserved populations on a sliding-fee scale for service, with some services subsidised by charity and/or governmental organizations.

### 2.2 Participant recruitment and data collection

We enrolled a convenience sample of pregnant women from October 2017 – January 2018. Women were approached for study participation, at any time in the pregnancy, when they presented to antenatal care (ANC) clinics at the study facilities for routine ANC visits. Study eligibility criteria included: age ≥ 15 years old, residents of the study counties and able to provide informed consent. Trained study personnel carried out interviews in English or local languages according to participant’s preference. Data were collected electronically using tablets. The questions used for this analysis were part of a broader survey assessing knowledge, attitudes and beliefs on maternal vaccines in general. We assessed pregnant women’s demographic characteristics and general obstetric history, knowledge, attitudes and beliefs on influenza and influenza vaccines. Questions on attitudes and beliefs were answered on a 3-point Likert scale (agree, neutral/no opinion and disagree). Questions on knowledge of influenza and attitudes towards influenza vaccination were answered as ‘yes’, ‘no’ or ‘not sure’. We also assessed pregnant women’s willingness to receive influenza vaccine if offered. The survey instrument was based upon previously used questionnaires shown to have high validity [24], [25], [26], [27] and the compendium of survey questions developed by the WHO SAGE working group on vaccine hesitancy [28]. The survey questionnaire was translated into local languages (Swahili, Luo, Gikuyu and Borana) and back-translated into English to ensure accuracy of translations before administration.

### 2.3 Data analysis

We analysed data using STATA version 13.0 (Stata Corp., College Station, TX) software package. We used descriptive statistics (counts, percentages, median, interquartile range) to describe quantitative and categorical study variables. We used chi-square and Fisher’s exact tests where appropriate to test for associations between socio-demographic characteristics and other survey variables. For women who answered to have heard of influenza before, we performed a binary logistic regression followed by multivariable logistic regression to identify variables associated with willingness to receive influenza vaccine in pregnancy. We compared those willing to be vaccinated with those who were not and with those who were unsure. Variables with *p*-value < 0.1 at bivariate level were included in multivariable logistic regression. Adjusted odds ratios (aORs) were determined with a *p*-value of < 0.05 considered significant and 95% confidence intervals (CIs) reported.

### 2.4 Ethical considerations

Ethical clearance for the study was obtained from KEMRI (SSC. 3292) and Emory University (IRB00089673) institutional review boards (IRBs), with CDC reliance on non-CDC IRB (CDC Protocol #6974.0). Written informed consent was obtained from all participants before enrolment.

## 3 Results

We enrolled 507 pregnant women from ANCs of participating facilities from October 2017-January 2018. The median age of participants was 26 (range 15–43) years; 55 (10.9%), 230 (45.4%), and 219 (43.2%) were in first, second and third trimester of pregnancy at the time of enrolment, respectively (Table 1). Almost half (n = 240) of women interviewed had primary school level or no education, 431 (85%) were married, 255 (50%) had some form of employment, and 328 (64.7%) lived in urban areas. Among 373 mothers in their second or more pregnancies, 102 (27.3%) reported having at least one prior miscarriage. The predominant ethnic group was Luo (n = 210, 41.4%), however these participants resided in different regions of the country: 96 (45.7%) from Siaya, 101 (48.1%) from Nairobi, 12 (5.7%) from Mombasa, and 1 (0.5%) from Marsabit).

**Table 1:** Socio-demographic and pregnancy characteristics of the women enrolled in the study, October 2017 – January 2018, N = 507.

Characteristic	N	%
*Site of study participation*		
Nairobi County	224	44.2
Mbagathi District Hospital	110	
Tabitha Clinic Kibera	114	
Mombasa County	104	20.5
Coast Provincial General Hospital	86	
Tudor Health Center	18	
Marsabit County	70	13.8
Marsabit District Hospital	70	
Siaya County	109	21.5
Siaya County Referral Hospital	101	
Lwak Mission Hospital	8	
*Maternal age*		
15–24 years	190	37.5
>24 years	317	62.5
Age (yrs), median (range)	26	(15,43)
*Gestational age*^a^		
First trimester	55	10.9
Second trimester	230	45.4
Third trimester	219	43.2
*Level of education*		
Below secondary	240	47.3
No Education	58	11.4
Primary Only	182	35.9
Secondary and above	267	52.7
Secondary	160	31.6
College	107	21.1
*Marital status*^b^		
Married	431	85.0
Not married	75	14.8
Single	70	93.3
Divorced/ Separated	4	5.3
Widow	1	1.3
*Primary source of income*		
Employed	255	50.3
Small business (no premise eg. sell maize)	85	33.3
Business owner (has premise eg. shop)	67	26.3
Salaried worker (eg. teacher, nurse, office)	50	19.6
Skilled labor (carpenter, tailor, artisan)	32	12.5
Unskilled labor (farming, construction)	21	8.2
Unemployed	252	49.7
Housewife	189	75.0
Not Working c	63	25.0
*Religion*		
Protestant	232	45.8
Catholic	127	25.1
Muslims	84	16.6
Traditional African Churches/traditional religion	64	12.6
*Ethnicity*		
Luo	210	41.4
Kikuyu/Embu/Meru/Mbeere	72	14.2
Borana/Rendile/Burji/Somali	67	13.2
Luhya/Teso	49	9.7
Swahilli/Mijikenda	52	10.3
Kamba	35	6.9
Other	22	4.3
*Type of residence*		
Urban	328	64.7
Peri/sub-urban	70	13.8
Rural	109	21.5
*No. of children living in the household*, median (IQR)	2(1,3)	
No. of children under < 5 years, median (IQR)	1 (1,1)	
*No. of pregnancies including the current one*		
1	134	26.4
2	161	31.8
3	112	22.1
≥4	100	19.7
Past miscarriages (mothers on 2nd or more pregnancies) d,	102	27.3
Hospitalization during current pregnancy	23	4.5

a Three women did not know their gestational age, n = 504

b One mother did not respond, n = 506

c Includes 35 mothers who reported being students and 8 who reported subsistence farming

d Responses excluded women in their first pregnancy, n = 373.

**Fig. 1:**
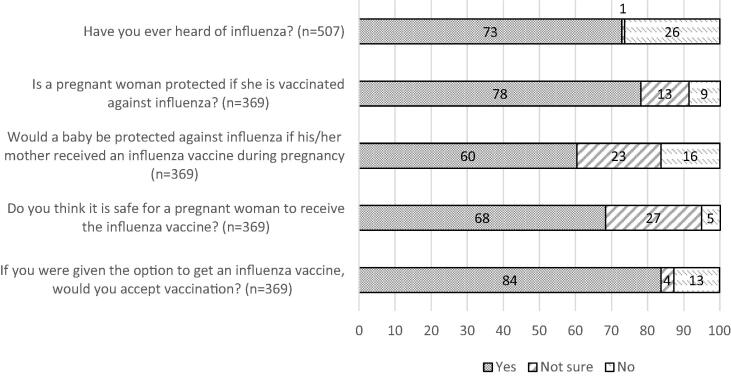
Proportions representing pregnant women’s knowledge and attitudes on influenza and influenza vaccination. Note: After question “Have you heard of influenza”, only those who said ‘yes’ were included in assessing their attitude towards influenza and influenza vaccination.

Overall, 369 (72.8%) women had heard about influenza (Fig. 1). Among those who had heard about influenza, 288 (78.1%) believed that a pregnant woman would be protected if she is vaccinated against influenza, 252 (68.3%) thought it was safe for a pregnant woman to receive the influenza vaccine, and 223 (60.4%) believed a baby would be protected against influenza if the mother received an influenza vaccine during pregnancy. Moreover, 309 (83.7%) women were willing to get an influenza vaccine if offered.

In bivariate analysis, willingness to accept influenza vaccine was associated with living in rural versus urban settings (OR 5.83; 95% CI 1.72, 19.70), belief that any maternal vaccines offered by government programs in the community are beneficial (OR 4.70; 95% CI 1.27, 17.32) and that a pregnant woman should be protected if she is vaccinated against influenza (OR 6.67; 95% CI 3.48, 12.79) (Table 2). Similarly, greater willingness to receive the vaccine was associated with belief that it is safe for a pregnant woman to receive influenza vaccine (OR 8.16; 95% CI 4.09, 16.27) and that the baby would be protected against influenza if mother received influenza vaccine during pregnancy (OR 2.37; 95% CI 1.27, 4.43). On the other hand, women > 24 years (OR 0.48; 95% CI 0.24, 0.98), general belief that there is no need for vaccines if diseases not common anymore (OR 0.43; 95% CI 0.23, 0.81), concern about serious adverse effects of vaccines in general (OR 0.46; 95% CI 0.25, 0.85) and that new vaccines carry more risks than older ones (OR 0.48; 95% CI 0.25, 0.92) were all associated with lower willingness to receive influenza vaccine. We did not observe any significant association between healthcare provider recommendation for vaccination in general and willingness to receive influenza vaccine.

**Table 2:** Associations between socio-demographic variables and attitudes towards influenza vaccine with willingness to receive influenza vaccine during pregnancy, Kenya^a^, N = 437.

	Willing to receive influenza vaccine (n = 384)		Unwilling to receive vaccine (n = 53)		Odd Ratios (95% CI)	p-value	Adjusted Odds Ratio	*p*-value
	n	%	n	%				
*Maternal age*								
15 – 24	120	39	11	23	Ref		Ref	
Over 24	189	61	36	77	**0.48 (0.24,0.98)**	**0.044**	0.54 (0.24,1.22)	0.140
*Level of education*								
Below Secondary	130	42	22	47	Ref			
Secondary & Above	179	58	25	53	1.21 (0.65,2.24)	0.541		
*Marital status*								
Not married	53	17	6	13	Ref			
Married	255	83	41	87	0.70 (0.28,1.74)	0.448		
*Primary source of income*								
Employment	172	56	26	55	Ref			
Unemployed	135	44	21	45	0.97 (0.52,1.80)	0.928		
*Religion*								
Catholic	82	27	10	21	Ref			
Protestant	148	48	22	47	0.82 (0.37,1.82)	0.630		
Muslim	33	11	6	13	0.67 (0.23,1.99)	0.473		
Other	46	15	9	19	0.62 (0.24,1.64)	0.334		
*Location of residence*								
Urban	139	45	30	64	Ref			
Peri/sub-urban	89	29	14	30	1.37 (0.69,2.73)	0.368	2.04 (0.78,5.31)	0.146
Rural	81	26	3	6	**5.83 (1.72,19.70)**	**0.005**	2.56 (0.66,9.97)	0.175
*Parity*								
Primiparous	86	28	11	23				
Multiparous	223	72	36	77	0.79 (0.39,1.63)	0.526		
*Miscarriage in the past*								
Yes	56	25	11	31	0.76 (0.35,1.65)	0.490		
No	167	75	25	69	Ref			
*Advised by healthcare worker to receive vaccine*								
Yes	194	63	30	64	0.96 (0.51,1.81)	0.890		
No	115	37	17	36	Ref			
*General attitudes towards vaccines*								
Perceived susceptibility								
I do not need vaccines for diseases that are not common anymore [Answered “Agree”]	80	26	21	45	**0.43 (0.23,0.81)**	**0.009**	0.55 (0.24,1.23)	0.144
Perceived benefit								
Getting vaccines is a good way to protect myself from disease	306	99	45	96	4.53 (0.74,27.88)	0.103	2.06 (0.26,16.17)	0.494
All maternal vaccines offered by the government program in my community are beneficial	303	98	43	91	**4.70 (1.27,17.32)**	**0.020**	1.94 (0.41,9.26)	0.404
Perceived barrier								
I am concerned about serious adverse effects of vaccines	100	32	24	51	**0.46 (0.25,0.85)**	**0.014**	0.61 (0.29,1.28)	0.194
New vaccines carry more risks than older vaccines	71	23	18	38	**0.48 (0.25,0.92)**	**0.026**	0.54 (0.24,1.21)	0.137
*Attitudes towards influenza vaccines*								
A pregnant woman is protected if she is vaccinated against influenza	257	83	20	43	**6.67 (3.48,12.79)**	**<0.001**	**3.87 (1.56,9.59)**	**0.003**
I think it is safe for a pregnant woman to receive influenza vaccine	234	76	13	28	**8.16 (4.09,16.27)**	**<0.001**	**5.32 (2.35,12.01)**	**<0.001**
Baby protected against flu if mother received an influenza vaccine during pregnancy	197	64	20	43	**2.37 (1.27,4.43)**	**0.010**	0.93 (0.42,2.05)	0.858

Variables with crude association showing p-value < 0.1 were included in the adjusted model.

a Comparison between mothers willing to receive influenza vaccine and those unwilling, among women who had heard about influenza.

Factors significantly associated with willingness to accept influenza vaccine in the multivariable model included the belief that “a pregnant woman is protected if she is vaccinated against influenza” (OR 3.87; 95% CI 1.56, 9.59) and a belief that the influenza vaccine is safe (OR 5.32; 95% CI 2.35, 12.01) (Table 2). Similar associations were observed when comparisons were made between mothers who were willing to and those who were not sure of receiving influenza vaccines (N = 13), however the predictors of willingness to receive influenza vaccine that remained significant was the belief that “all maternal vaccines offered by the government program in my community are beneficial” (OR 12.98; 95% CI 2.46, 68.43) and the belief that influenza vaccines should be safe if given during pregnancy (OR 4.89; 95% CI 1.45, 16.48) (Supplemental table).

## 4 Discussion

Approximately 70% of Kenyan pregnant women interviewed in our study had heard about influenza. Among those, most believed that they would be protected (78.1%) if they received the influenza vaccine, felt that it was safe (68.3%) to receive the vaccine during pregnancy and that their baby would also be protected (60.4%) if the mother received influenza vaccine while pregnant. Belief in protective effect of influenza vaccine and its safety were independent predictors of willingness to receive influenza vaccine among Kenyan pregnant women. To the best of our knowledge, this is the first study to assess awareness of influenza, and beliefs and attitudes towards influenza vaccination in a large group of pregnant women in tropical Africa.

Comparing our results with studies in countries where the government already recommends influenza vaccine during pregnancy, Kenyan women had more confidence on the effectiveness and safety of influenza vaccines. For instance, a US study assessing factors associated with intention to receive influenza, tetanus, diphtheria, and acellular pertussis vaccines during pregnancy reported that half of surveyed pregnant women had concerns about vaccine safety [29]. In a cross-sectional survey in Pennsylvania assessing patients’ perception and acceptability of vaccines during pregnancy, 61% of pregnant women who participated in the survey reported safety concerns and 8% believed that influenza vaccine caused influenza [30]. In Hong Kong, where seasonal influenza vaccine is not available in publicly funded antenatal clinics but is subsidized for low-income women, substantial concerns on potential side effects of influenza vaccine to the foetus (63%) and pregnant woman (53%) were reported among pregnant women who did not receive influenza vaccine [22]. In China, influenza vaccine is offered at expanded program on immunization (EPI) clinics but is not part of the national schedule; Chinese pregnant women raised safety concerns on influenza vaccine to their foetus (83%) and for themselves (28%), and 22% of the women believed the vaccine was not necessary [31]. Tetanus vaccine is currently the only vaccine recommended during pregnancy in Kenya and provided free of charge. It is difficult to predict if the high level of vaccine confidence observed among pregnant women in our study would translate into actual acceptability of the influenza vaccine if offered. Nevertheless, such high levels of vaccine confidence are critical for acceptance of existing vaccination programs and introduction of new ones in face of the growing global problem of vaccine hesitancy [32].

We observed in our study that approximately 30% of the pregnant women reported not having heard of influenza. Awareness of the burden of influenza disease during pregnancy and the impact it could have on pregnancy outcome and on new-borns as well as information on the benefit afforded by vaccinating pregnant women are vital when considering rolling out a maternal influenza vaccination program. Several studies on maternal influenza vaccination have demonstrated that maternal knowledge of influenza disease and vaccines together with attitudes and beliefs are important determining factors in influenza vaccine uptake [23], [33], [34]. While these studies are most concentrated in high-income countries, very little is known on knowledge, attitudes and beliefs held by pregnant women in low-middle-income countries where influenza vaccines are not routinely offered by governments.

In our study, a substantial proportion (83.7%) of women who knew about influenza agreed that they would accept influenza vaccine during pregnancy if given an option. The expressed willingness by the women to receive influenza vaccine could suggest a high acceptability in the future if influenza vaccination is implemented in Kenya. Nonetheless, this high acceptability seen in our study contrasts with others from high-income countries that reported low acceptance of maternal influenza vaccine despite the vaccine being offered by the government. For instance, 42% of pregnant women who were familiar with influenza vaccine reported willingness to receive the vaccine in Thailand [35]. In Germany, 11% of pregnant women who never received influenza vaccination reported being willing to be vaccinated, despite influenza vaccine being recommended for pregnant women in their country [36], while 34% of pregnant women in the state of Georgia, USA, reported intentions to receive the 2012/2013 influenza vaccine [29].

Independent predictors of willingness to be vaccinated were the belief that the influenza vaccines are effective and safe for pregnancy. Public health messages focusing on the effectiveness of the influenza vaccine in protecting mothers and babies [37] and the long safety profile of influenza vaccine among this group [22] may help avoid hesitancy when the program is implemented in Kenya. Of note, another important predictor of willingness to accept vaccination when comparisons were made with mothers who were not sure of receiving the vaccine was the belief that any maternal vaccines offered by the government program in the community are beneficial. This supports findings from other studies that have shown public trust to be directly associated with vaccine acceptance [25], and shows the importance of government engagement on vaccine recommendations and target public health messages. Much as we did not observe any significant association between healthcare provider recommendation for vaccination in general and willingness to receive influenza vaccine, ANC settings with government healthcare providers still represent an opportunity to provide women with information on influenza vaccines. To capitalize on this opportunity and raise awareness levels of influenza and influenza vaccines among pregnant women in Kenya and in other countries planning adoption of influenza vaccination in pregnancy, healthcare providers are best placed to communicate influenza vaccine information [36], [38], [39], [40].

This study had some limitations. First, this was a convenience sample and we did not capture participation rates, neither did we collect sociodemographic data on all the pregnant women from our study sites during the study period, this limited our ability to assess the representability of our convenience sample. Among respondents, 41% were of Luo ethnicity. Nonetheless, the respondents lived in different regions of the country and beliefs and practices could be influenced by cultures and lifestyle from their place of residence. The study was conducted in four of the 47 counties in Kenya and the findings may not be generalizable to all the counties, despite the attempt to select counties with diverse geographical settings and cultures (i.e., western, central, northern and coastal regions of Kenya). Another aspect is that the study was conducted mostly among women attending governmental health facilities and this may represent a group in the population who trust government-funded health sector and their health policy recommendations. Finally, although most women reported willingness to receive influenza vaccine, it may not be a reliable indicator of actual vaccination behaviour.

## 5 Conclusion

Approximately one third of pregnant women interviewed had never heard of influenza. Willingness to be vaccinated against influenza while pregnant if given the opportunity was higher among those familiar with the disease, despite the vaccine not being offered by the government. Government recommendation for maternal influenza vaccination in Kenya may lead to high rates of vaccine acceptance because of the overall trust placed in the government vaccination programs. Nonetheless, strategies for improving influenza vaccine acceptance may aim at improving overall knowledge of influenza among pregnant women, mitigating safety concerns, and educating hesitant mothers on the benefits of vaccinating during pregnancy for themselves and their new-borns.

## Author contributions

NAO participated in the design of the study, study implementation oversight, data analysis and interpretation and wrote the manuscript. WNW, RA collected data and contributed to interpretation of study findings. FO, BN, MA, contributed to data analysis and interpretation. ADW participated in the design of the study, study implementation oversight and interpretation of findings. IGC, FAM, MAW, JRV contributed to interpretation of findings. SBO proposed the study, contributed to the study design, and interpretation of findings. SSC participated in the design of the study, data analysis, interpretation and writing of the manuscript.

All authors reviewed and approved the submitted manuscript.

## Declaration of Competing Interest

The authors declare that they have no known competing financial interests or personal relationships that could have appeared to influence the work reported in this paper.
