# Supplementary material for: Knowledge and attitudes towards influenza and influenza vaccination among pregnant women in Kenya
Source: Vaccine. 2020 Oct 7;38(43):6832–8. doi: 10.1016/j.vaccine.2020.08.015 (PMC7526973; doi:10.1016/j.vaccine.2020.08.015)
Supplement: Supplementary data 1 [file mmc1.docx]

Supplemental Table. Associations between socio-demographic variables and attitudes towards influenza vaccine with willingness to receive influenza vaccine in pregnant women in Kenya^a^, N=397

|  | | Willing to receive influenza vaccine (n= 384) | | Not sure  (n= 13) | | | Odd Ratios (95% CI) | | p-value | | | Adjusted OR | | *p*-value | | |  |  |
| --- | --- | --- | --- | --- | --- | --- | --- | --- | --- | --- | --- | --- | --- | --- | --- | --- | --- | --- |
|  | n | | % | n | % |  | | |  |  | | | | | |  | | |
| Maternal age |  | |  |  |  |  | | |  |  | | | | | |  | | |
| 15 - 24 | 120 | | 39 | 1 | 8 | Ref | |  | | | Ref | |  | | | |  |  |
| Over 24 | 189 | | 61 | 12 | 92 | 0.13 (0.02,1.02) | | 0.053 | | | 0.13 (0.02,1.03) | | | | 0.054 | |  |  |
|  |  | |  |  |  |  | |  | | |  | |  | | | |  |  |
| Level of education |  | |  |  |  |  | |  | | |  | |  | | | |  |  |
| Below Secondary | 130 | | 42 | 4 | 31 | Ref | |  | | |  | |  | | | |  |  |
| Secondary & Above | 179 | | 58 | 9 | 69 | 0.61 (0.18,2.03) | | 0.422 | | |  | |  | | | |  |  |
|  |  | |  |  |  |  | |  | | |  | |  | | | |  |  |
| Marital status |  | |  |  |  |  | |  | | |  | |  | | | |  |  |
| Not married | 53 | | 17 | 12 | 92 | Ref | |  | | |  | |  | | | |  |  |
| Married | 255 | | 83 | 1 | 8 | 0.40 (0.05,3.15) | | 0.385 | | |  | |  | | | |  |  |
|  |  | |  |  |  |  | |  | | |  | |  | | | |  |  |
| Primary source of income |  | |  |  |  |  | |  | | |  | |  | | | |  |  |
| Employment | 172 | | 56 | 10 | 77 | Ref | |  | | |  | |  | | | |  |  |
| Unemployed | 135 | | 44 | 3 | 23 | 2.62 (0.71,9.69) | | 0.150 | | |  | |  | | | |  |  |
|  |  | |  |  |  |  | |  | | |  | |  | | | |  |  |
| Religion |  | |  |  |  |  | |  | | |  | |  | | | |  |  |
| Catholic | 82 | | 27 | 5 | 38 | Ref | |  | | |  | |  | | | |  |  |
| Protestant | 148 | | 48 | 6 | 46 | 1.50 (0.45,5.08) | | 0.511 | | |  | |  | | | |  |  |
| Muslim | 33 | | 11 | 1 | 8 | 2.01 (0.23,17.88) | | 0.531 | | |  | |  | | | |  |  |
| Other | 46 | | 15 | 1 | 8 | 2.80 (0.32,24.74) | | 0.353 | | |  | |  | | | |  |  |
|  |  | |  |  |  |  | |  | | |  | |  | | | |  |  |
| Location of residence |  | |  |  |  |  | |  | | |  | |  | | | |  |  |
| Urban | 139 | | 45 | 11 | 85 | Ref | |  | | |  | |  | | | |  |  |
| Peri/sub-urban | 89 | | 29 | 0 | 0 |  | |  | | |  | |  | | | |  |  |
| Rural | 81 | | 26 | 2 | 15 | 3.21 (0.69,14.82) | | 0.136 | | |  | |  | | | |  |  |
|  |  | |  |  |  |  | |  | | |  | |  | | | |  |  |
| Parity |  | |  |  |  |  | |  | | |  | |  | | | |  |  |
| Primiparous | 86 | | 28 | 3 | 23 |  | |  | | |  | |  | | | |  |  |
| Multiparous | 223 | | 72 | 10 | 77 | 0.78 (0.21,2.89) | | 0.708 | | |  | |  | | | |  |  |
|  |  | |  |  |  |  | |  | | |  | |  | | | |  |  |
| Miscarriage in the past |  | |  |  |  |  | |  | | |  | |  | | | |  |  |
| Yes | 56 | | 25 | 1 | 10 | 3.02 (0.37,24.35) | | 0.300 | | |  | |  | | | |  |  |
| No | 167 | | 75 | 9 | 90 | Ref | |  | | |  | |  | | | |  |  |
|  |  | |  |  |  |  | |  | | |  | |  | | | |  |  |
| Adviced by healthcare worker to receive vaccine |  | |  |  |  |  | |  | | |  | |  | | | |  |  |
| Yes | 194 | | 63 | 7 | 54 | 1.45 (0.47,4.41) | | 0.517 | | |  | |  | | | |  |  |
| No | 115 | | 37 | 6 | 46 | Ref | |  | | |  | |  | | | |  |  |
|  |  | |  |  |  |  | | |  |  | | | | | |  | | |
| General attitudes towards vaccines |  | |  |  |  |  | | |  |  | | | | | |  | | |
| Perceived susceptibility |  | |  |  |  |  | | |  |  | | | | | |  | | |
| I do not need vaccines for diseases that are not common anymore [Answered "YES"] | 80 | | 26 | 4 | 31 | 0.79 (0.24,2.62) | | | 0.695 | 0.51 (0.14,1.89) | | | | | 0.316 | | |  |
|  |  | |  |  |  |  | | |  |  | | | | |  | | |  |
| Perceived benefit |  | |  |  |  |  | | |  |  | | | | |  | | |  |
| Getting vaccines is a good way to protect myself from disease | 381 | | 99 | 16 | 100 |  | | |  |  | | | | |  | | |  |
| Maternal vaccines are effective | 298 | | 96 | 12 | 92 | 2.26 (0.27,18.94) | | | 0.450 |  | | | | |  | | |  |
| Vaccines given in pregnancy are important for my health | 307 | | 99 | 12 | 92 | **12.79 (1.08,151.04)** | | | **0.043** |  | | | | |  | | |  |
| All maternal vaccines offered by the government program in my community are beneficial | 303 | | 98 | 10 | 77 | **15.15 (3.31,69.43)** | | | **0.001** | **12.98 (2.46,68.43)** | | | | | **0.003** | | |  |
|  |  | |  |  |  |  | | |  |  | | | | |  | | |  |
| Perceived barrier |  | |  |  |  |  | | |  |  | | | | |  | | |  |
| I am concerned about serious adverse effects of vaccines | 100 | | 32 | 6 | 46 | 0.56 (0.18,1.70) | | | 0.306 |  | | | | |  | | |  |
| New vaccines carry more risks than older vaccines | 71 | | 23 | 2 | 15 | 1.64 (0.36,7.58) | | | 0.526 |  | | | | |  | | |  |
|  |  | |  |  |  |  | | |  |  | | | | |  | | |  |
| Attitudes towards influenza vaccines |  | |  |  |  |  | | |  |  | | | | |  | | |  |
| I think that a pregnant woman should be vaccinated against influenza | 257 | | 83 | 6 | 46 | **5.77 (1.86,17.86)** | | | **0.002** |  | | | | |  | | |  |
| It is likely for a pregnant woman who was not been vaccinated against influenza to contract the disease | 227 | | 73 | 4 | 31 | **6.23 (1.87,20.77)** | | | **0.003** |  | | | | |  | | |  |
| A pregnant woman is protected if she is vaccinated against influenza | 257 | | 83 | 11 | 85 | 0.90 (0.19,4.17) | | | 0.892 |  | | | | |  | | |  |
| I think it is safe for a pregnant woman to receive influenza vaccine | 234 | | 76 | 5 | 38 | **4.99 (1.59,15.72)** | | | **0.006** | **4.89 (1.45,16.48)** | | | | | **0.010** | | |  |
| Baby protected against flu if mother received an influenza vaccine during pregnancy | 197 | | 64 | 6 | 46 | 2.05 (0.67,6.26) | | | 0.206 |  | | | | | |  | | |

^a^Comparison between mothers willing to receive influenza vaccine and those not sure, among women who had heard about influenza.

Variables with crude association showing *p*-value <0.1 were included in the adjusted model.
